# Treatment-Stratified Immune–Inflammatory Trajectories and Early Response in Patients with Hepatocellular Carcinoma Receiving Systemic Therapy

**DOI:** 10.3390/diagnostics16203329

**Published:** 2026-10-13

**Authors:** Ashley Kim, Manaf Alsudaney, Osama Khattab, Daniel Legaspi, Naomy Kim, Shahzeb Imran, Angelica E. Alderete, Pin-Jung Chen, Fadhil Jamil, Jason Liu, Jihye Lim, Ekaterina Koltsova, Walid Ayoub, Andrew Hendifar, Ju Dong Yang

**Affiliations:** 1 Department of Medicine, Karsh Division of Gastroenterology and Hepatology, Comprehensive Transplant Center, Cedars-Sinai Health Sciences University, Los Angeles, CA 90048, USA; ashley.kim7@cshs.org (A.K.); osama.khattab@cshs.org (O.K.); daniel.legaspi@cshs.org (D.L.); naomy.kim@cshs.org (N.K.); walid.ayoub@cshs.org (W.A.); judong.yang@cshs.org (J.D.Y.); 2 Comprehensive Transplant Center, Cedars-Sinai Health Sciences University, Los Angeles, CA 90048, USA; 3 School of Medicine, Dentistry & Biomedical Sciences, Queen’s University Belfast, Belfast BT7 1NN, UK; simran02@qub.ac.uk; 4 Department of Enterprise Research Admin, Academic Affairs, Cedars-Sinai Health Sciences University, Los Angeles, CA 90048, USA; angelica.alderete@cshs.org; 5 Samuel Oschin Comprehensive Cancer Institute, Cedars-Sinai Health Sciences University, Los Angeles, CA 90048, USA; pinjung.amy.chen@gmail.com (P.-J.C.); andrew.hendifar@csmc.edu (A.H.); 6 Basic Medical Science, College of Medicine, American University of Antigua, St. John’s P.O. Box W1451, Antigua and Barbuda; jamil.fadhil@gmail.com; 7 Chicago Medical School, Rosalind Franklin University of Medicine and Science, North Chicago, IL 60064, USA; jason.liu@my.rosalindfranklin.edu; 8 The Catholic University Liver Research Center, College of Medicine, The Catholic University of Korea, Seoul 06591, Republic of Korea; limbecca@hanmail.net; 9 Department of Internal Medicine, Yeouido St. Mary’s Hospital, College of Medicine, The Catholic University of Korea, Seoul 07345, Republic of Korea; 10 Cedars-Sinai Cancer, Department of Medicine, Cardiology and Biomedical Sciences, Cedars-Sinai Health Sciences University, Los Angeles, CA 90048, USA; ekaterina.koltsova@csmc.edu

**Keywords:** hepatocellular carcinoma, systemic therapy, systemic immune-inflammation index, immune–inflammatory trajectories, treatment response, biomarker discovery, molecular diagnostics

## Abstract

**Background:** Systemic therapy outcomes in hepatocellular carcinoma (HCC) remain heterogeneous, and treatment selection is still largely guided by clinical eligibility rather than dynamic biologic response. We evaluated whether accessible standard-of-care immunophenotypic and immune–inflammatory markers, tumor-marker kinetics, and immune-context variables could generate treatment-stratified early response signals. **Methods:** We conducted a retrospective analysis of 153 unique patients with HCC, each contributing one systemic therapy episode. First on-treatment imaging response was classified as complete response, partial response, stable disease, or progressive disease. Routine immune-cell indices, including neutrophil-to-lymphocyte ratio, platelet-to-lymphocyte ratio, monocyte-to-lymphocyte ratio, lymphocyte-to-monocyte ratio, and systemic immune-inflammation index (SII), were evaluated at baseline and early post-treatment timepoints. Alpha-fetoprotein (AFP) kinetics and overall survival were analyzed descriptively. **Results:** A total of 153 patients were included: 124 received immunotherapy-based therapy and 29 received tyrosine kinase inhibitor (TKI) therapy. Overall objective response rate was 26.8%, disease control rate was 56.2%, and progression occurred in 43.8%. AFP trajectory separated responder from non-responder phenotypes, supporting the value of dynamic marker change. Treatment-stratified trajectories revealed distinct patterns: among immunotherapy-treated patients, Week 2 SII increase was associated with progression at first on-treatment imaging (median log2 ratio 0.17 vs. −0.24 for progression vs. non-progression; *p* = 0.026), whereas TKI response was associated with lymphocyte expansion and decline in neutrophil-dominant inflammatory indices. **Conclusions:** Routine immune–inflammatory trajectories, particularly early SII change, may provide clinically relevant treatment-stratified response signals in patients with HCC receiving systemic therapy. These findings support interpreting standard clinical markers as evolving biologic trajectories rather than isolated prognostic scores and provide a practical foundation for future biomarker-informed treatment strategies.

## 1. Introduction

**Figure 1:**
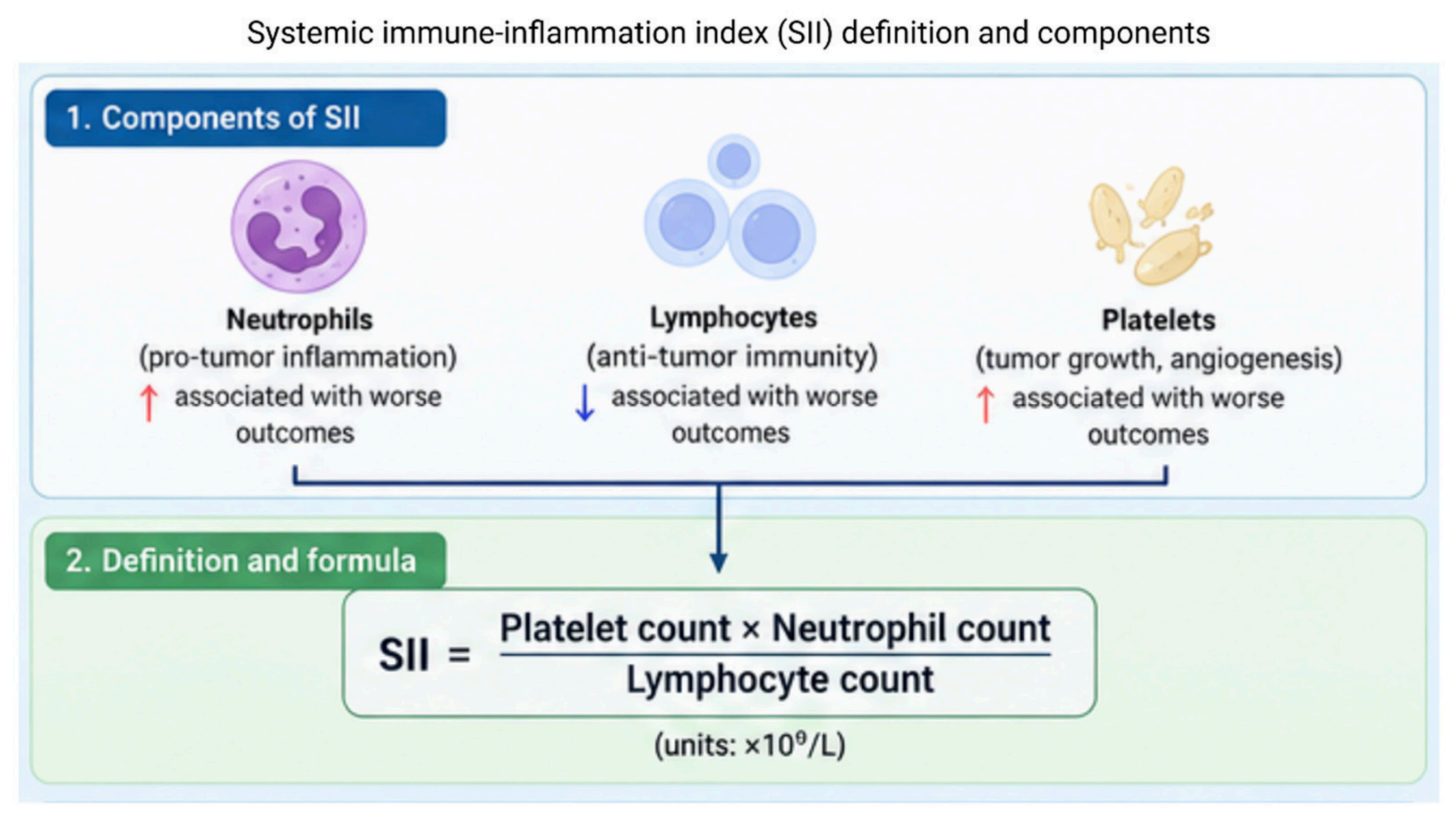
Illustrative overview of the systemic immune-inflammation index (SII). SII is calculated as platelet count × neutrophil count/lymphocyte count and reflects the balance between neutrophil- and platelet-associated inflammation and lymphocyte-mediated immune surveillance. Created with BioRender; limited graphical elements were prepared using AI-assisted features. All scientific content and final figure composition were reviewed by the authors.

Hepatocellular carcinoma (HCC) remains a leading cause of cancer-related mortality and commonly develops in the setting of chronic liver disease, cirrhosis, and persistent immune–inflammatory remodeling [1,2,3]. In this context, immune–inflammatory remodeling refers to sustained changes in the hepatic immune environment, including chronic inflammatory signaling, altered innate and adaptive immune-cell activity, and progressive impairment of antitumor immune surveillance. The systemic treatment landscape for unresectable or advanced HCC has expanded substantially, with immune checkpoint inhibitors (ICIs), anti-vascular endothelial growth factor (VEGF)-based combinations, dual checkpoint blockade, and tyrosine kinase inhibitors (TKIs) now forming the backbone of therapy [4,5,6]. However, clinical benefit remains heterogeneous, and treatment selection is still largely guided by clinical eligibility, liver function, tumor burden, and contraindications rather than by a validated biologic framework that predicts which therapy is most likely to benefit an individual patient. Because chronic liver injury and cirrhosis shape systemic inflammation, immune surveillance, myeloid–lymphocyte balance, and the tumor microenvironment, early on-treatment biologic trajectories may provide clinically relevant response signals that complement baseline clinical and laboratory markers [3,7]. Current response assessment and biomarker strategies only partially address this need. Modified Response Evaluation Criteria in Solid Tumors (mRECIST) provides a radiographic framework for assessing viable tumor burden, but imaging response is usually available only after several weeks of treatment [8]. Alpha-fetoprotein (AFP) is a practical circulating tumor marker, and early AFP decline has been associated with favorable outcomes across systemic therapy settings [9,10]. Inflammatory and tumor-marker combinations, such as C-reactive protein and AFP in immunotherapy (CRAFITY), and other inflammatory biomarker studies further support the clinical relevance of host inflammation and tumor-marker kinetics [11,12]. However, these approaches do not fully establish whether baseline immune context and early immune–inflammatory trajectories can be interpreted in different treatment contexts across different systemic therapy mechanisms. Routine blood-count-derived immune trajectories may serve as accessible immunophenotypic biomarkers that complement molecular diagnostics by providing a longitudinal view of the systemic host–tumor immune state using measurements already available in clinical practice. Although these indices do not directly measure genomic or transcriptomic alterations, they may provide a practical phenotypic layer linking routine clinical assessment with deeper molecular characterization of treatment response and resistance. The neutrophil-to-lymphocyte ratio (NLR), platelet-to-lymphocyte ratio (PLR), monocyte-to-lymphocyte ratio, lymphocyte-to-monocyte ratio, and systemic immune-inflammation index (SII) reflect different aspects of systemic inflammatory balance. SII, calculated as platelet count × absolute neutrophil count/absolute lymphocyte count, integrates platelet-associated inflammation and angiogenesis, neutrophil/myeloid inflammatory activity, and lymphocyte-mediated immune reserve into a single accessible marker [13]. The components and calculation of SII are illustrated in Figure 1.

Prior studies have evaluated SII as a prognostic marker in unresectable HCC treated with immune-based therapy, and emerging work has suggested that changes in SII may predict outcomes in selected treatment settings such as lenvatinib plus programmed cell death protein 1 (PD-1) inhibition [13,14,15]. Thus, while SII itself is not a novel formula, its longitudinal treatment-context behavior remains incompletely defined. Autoimmunity-related markers may provide an additional layer of immune-context information. Antinuclear antibody (ANA) positivity and other autoantibodies have been studied in cancer and ICI-treated populations, largely in relation to immune-related adverse events [16,17,18]. In HCC and cirrhosis, autoantibody patterns may also reflect broader immune dysregulation or immune surveillance phenotypes [17,18,19]. In this study, ANA was evaluated not as a toxicity marker, but as an exploratory immune-context variable that may modify response patterns. This is especially relevant because retrospective standard-of-care (SOC) testing is incomplete, whereas future studies can standardize ANA and smooth muscle antibody assessment. Despite growing interest in inflammatory biomarkers, important gaps remain: most studies emphasize baseline prognostic markers rather than early dynamic trajectories; many focus on a single treatment class or regimen; and retrospective SOC datasets often lack direct mechanistic biomarkers such as interleukin-6, high-sensitivity C-reactive protein, C-X-C motif chemokine ligands 9 and 10, VEGF-A, soluble VEGF receptor 2, fibroblast growth factor 19/23, immune-cell signaling assays, and proteomic profiling [7,8,9,10,11,12,15,20]. We therefore designed this retrospective signal-discovery analysis to identify accessible immunophenotypic trajectory signals associated with systemic therapy response and to define candidates for subsequent integration with molecular and mechanistic response biomarkers. We hypothesized that early immune–inflammatory trajectories would differ by treatment mechanism and response phenotype, helping identify which routine signals are clinically useful and which require deeper prospective validation.

## 2. Methods

### 2.1. Study Design and Cohort

We conducted a retrospective signal-discovery analysis of the HCC-HYB-01 (IRB: STUDY00004803) retrospective arm to evaluate whether routinely available immune-related clinical variables and early on-treatment trajectories could generate treatment-stratified response signals in patients receiving systemic therapy for HCC. The analytic cohort included 153 unique patients, each contributing one systemic therapy episode, with available treatment regimen, first on-treatment imaging response, and routine clinical follow-up data. No patient contributed more than one treatment episode to this analysis. All index treatment episodes represented first-line systemic therapy for HCC, and no patient had received prior systemic HCC therapy before Day 0. Subsequent systemic regimens initiated after the index treatment were captured longitudinally within the same patient record but were not analyzed as separate index treatment episodes. Therapies were grouped into two broad treatment classes: immunotherapy-based regimens and TKI therapy. Regimen-level categories included atezolizumab/bevacizumab, durvalumab/tremelimumab, nivolumab, lenvatinib, sorafenib, pembrolizumab, atezolizumab alone, durvalumab alone, and regorafenib. Regimen-level analyses were considered exploratory because subgroup sizes were small. The study was approved by the Cedars-Sinai Institutional Review Board (STUDY00004803) and was conducted in accordance with applicable human-subject research standards. The requirement for informed consent was waived by the Institutional Review Board for this retrospective analysis.

### 2.2. Outcomes

The primary outcome was radiographic response at first on-treatment imaging. Response categories included complete response (CR), partial response (PR), stable disease (SD), and progressive disease (PD). Objective response rate (ORR) was defined as CR plus PR, and disease control rate (DCR) was defined as CR plus PR plus SD. Progression at first on-treatment imaging was defined as PD at first on-treatment imaging. First on-treatment imaging was obtained as part of routine clinical care rather than according to a study-mandated imaging schedule. The interval from systemic therapy initiation to first on-treatment imaging was calculated for each patient and summarized overall and by treatment class. Week 2, Week 6, and Week 12 laboratory measurements represented standard-of-care values assigned to the corresponding prespecified time windows. To evaluate the temporal relationship between biomarker assessment and radiographic response, each laboratory window was classified as occurring entirely before first imaging, overlapping the imaging date, or occurring after first imaging. Sensitivity analyses of the principal Week 2 SII finding were also performed after restricting first on-treatment imaging to clinically relevant timing windows. Protocol-defined paradoxical flare was defined as an early worsening pattern at first on-treatment imaging, consisting of ≥25% increase in mRECIST-defined tumor burden from baseline together with ≥50% increase in serum IL-6 from baseline to the same imaging timepoint. Because the retrospective cohort relied on standard-of-care laboratory and imaging data, serial IL-6 was not routinely available and protocol-defined paradoxical flare could not be confirmed retrospectively. Therefore, as an exploratory descriptive variable among patients with PD at first on-treatment imaging, we evaluated imaging-only tumor-burden increase ≥25%, defined as ≥25% increase in mRECIST-defined tumor burden from baseline. This variable was analyzed only as a subset of PD and was not considered a separate response category or confirmed inflammatory flare. Overall survival (OS) was evaluated from the date of systemic therapy start till last follow up or death.

#### 2.2.1. Routine Immune-Cell Indices and Biochemical Trajectories

Routine blood-count-derived immune variables included absolute neutrophil count, absolute lymphocyte count, monocyte count, platelet count, NLR, PLR, monocyte-to-lymphocyte ratio, lymphocyte-to-monocyte ratio, and SII. SII was calculated as platelet count × absolute neutrophil count/absolute lymphocyte count. These variables were evaluated at baseline and available post-treatment timepoints, including Week 2, Week 6, and Week 12. AFP, albumin, bilirubin, and available hsCRP values were also evaluated as routine clinical or inflammatory markers. For longitudinal analyses, post-treatment change was summarized using log2 ratio to baseline, calculated as log2 (post-treatment value/baseline value) when baseline and follow-up values were available. A log2 ratio greater than 0 indicated an increase from baseline, while a value less than 0 indicated a decline.

#### 2.2.2. Autoimmunity and Immune-Status Variables

ANA status was evaluated as an exploratory autoimmunity-related variable. Episodes were categorized as ANA-positive, ANA-negative, or unknown/not tested. ANA titer was further grouped as negative, low positive (1:40), moderate/high positive (≥1:80), or unknown/not tested. Because ANA testing was not standardized in the retrospective SOC dataset, ANA analyses were interpreted as hypothesis-generating and were not used to infer true absence of autoimmunity among untested episodes. HIV status was evaluated descriptively as an immune-status variable. Because the HIV-positive subgroup was small, analyses by HIV status were interpreted as descriptive and exploratory.

#### 2.2.3. Statistical Analysis

Categorical variables were summarized as counts and percentages. Continuous variables and trajectory measures were summarized using medians and interquartile ranges (IQR). Treatment response outcomes were summarized overall, by treatment class, and by regimen. Comparisons of continuous immune-cell, biochemical, and trajectory variables between outcome groups were performed using non-parametric rank-based testing. Fisher exact testing was used for small categorical comparisons, including exploratory HIV-status analyses. OS was summarized using survival curves and compared by progression status at first on-treatment imaging status using log-rank testing. Exploratory treatment-by-marker interaction analyses were performed using logistic regression models including treatment class, the continuous biomarker log2 ratio, and a treatment class × biomarker interaction term. Separate models were evaluated for progression at first on-treatment imaging and objective response, as appropriate to the trajectory of interest. Interaction effect estimates are reported as interaction odds ratios with 95% confidence intervals and *p*-values. Given the exploratory signal-discovery design and relatively small TKI subgroup, these analyses were interpreted cautiously and were not adjusted for multiplicity. Within the immunotherapy cohort, the Week 2 SII–progression association was additionally examined across atezolizumab/bevacizumab, durvalumab/tremelimumab, and pooled single-agent PD-1/PD-L1 therapy; regimen-stratified comparisons and an exploratory regimen × SII interaction analysis were performed. Given that platelet count is a component of SII and may also reflect underlying liver disease severity, sensitivity analyses of the principal Week 2 SII–progression association were performed using logistic regression with adjustment for baseline Child–Pugh score. Additional sensitivity models accounted for baseline platelet count or MELD-Na, and the analysis was repeated after restriction to patients with cirrhosis. Regimen-level and ANA subgroup findings were considered hypothesis-generating because subgroup sizes were small.

## 3. Results

### 3.1. Patient Characteristics

The cohort included 153 unique patients, each represented by one first-line systemic therapy episode; 124 received immunotherapy-based treatment and 29 received TKI therapy. All index episodes were first-line treatment (153/153, 100%), including 100% of patients within each individual regimen. Baseline patient, liver disease, tumor-burden, and immune-context characteristics are summarized in Table 1. Overall, the cohort was predominantly male, most patients had cirrhosis, and most had intermediate or advanced BCLC stage disease. Baseline routine immune–inflammatory markers were similar between treatment classes, supporting the exploratory nature of subsequent treatment-stratified trajectory analyses.

### 3.2. Factors Associated with Treatment Response and Overall Survival

Overall, ORR was 41/153 (26.8%), DCR was 86/153 (56.2%), and PD at first on-treatment imaging occurred in 67/153 (43.8%). Among 67 patients with PD at first on-treatment imaging, 24 (35.8%) had imaging-only tumor-burden increase ≥25%. All cases meeting this imaging-only criterion were contained within the broader PD category. At the broad treatment class level, immunotherapy-based episodes had a numerically higher ORR than TKI episodes, while TKI episodes had a higher DCR. Formal treatment-by-marker interaction analyses provided evidence of treatment class differences for selected, but not all, trajectories. For the principal Week 2 SII association with progression, the treatment class × SII interaction was not significant (interaction OR, 1.17; 95% CI, 0.44–3.09; *p* = 0.757), indicating that the observed immunotherapy-stratified association should not be interpreted as definitive evidence of a differential treatment effect. In contrast, treatment class interactions were significant for Week 12 neutrophil change (interaction OR, 0.084; 95% CI, 0.008–0.903; *p* = 0.041) and Week 12 NLR change (interaction OR, 0.172; 95% CI, 0.031–0.962; *p* = 0.045) in relation to objective response. The Week 6 lymphocyte interaction did not reach statistical significance (*p* = 0.107), while the Week 12 SII interaction showed a nonsignificant trend (*p* = 0.086). Together, these analyses support treatment class differences for selected response-associated inflammatory trajectories but not a uniform treatment-stratified effect across biomarkers. The Week 2 SII association remained consistent after accounting for hepatic reserve. In a logistic regression model adjusted for baseline Child–Pugh score, higher Week 2 SII change remained associated with progression (adjusted OR, 1.58; 95% CI, 1.04–2.41; *p* = 0.034). The association was also preserved after additional adjustment for baseline platelet count (adjusted OR, 1.65; 95% CI, 1.07–2.55; *p* = 0.025). In a sensitivity analysis restricted to immunotherapy-treated patients with cirrhosis (*n* = 91), Week 2 SII remained higher among patients with PD than among those without PD (median log2 ratio, 0.41 vs. −0.24; *p* = 0.024), and the association remained after adjustment for Child–Pugh score (adjusted OR, 1.63; 95% CI, 1.03–2.57; *p* = 0.036). In an exploratory logistic model, each one-unit increase in the Week 2 SII log2 ratio was associated with higher odds of progression (OR, 1.55; 95% CI, 1.02–2.34; *p* = 0.038). Regimen-level response patterns varied, with the highest observed ORR in the durvalumab/tremelimumab group and the lowest ORR in the sorafenib group. These regimen-level results should be interpreted descriptively because treatment assignment was not randomized and subgroup sizes varied substantially (Table 2, Supplementary Figure S1).

First on-treatment imaging response provided a clinically meaningful outcome anchor for the retrospective analysis. The median interval from systemic therapy initiation to first on-treatment imaging was 79 days (IQR, 60–101) overall, 79 days (IQR, 61–99) for immunotherapy-based therapy, and 79 days (IQR, 55–111) for TKI therapy. Imaging was obtained according to routine clinical care rather than a standardized study-mandated schedule. Among patients with available Week 6 laboratory measurements, the Week 6 assessment window occurred entirely before first imaging in 119/142 (83.8%), whereas the Week 12 window occurred entirely before imaging in only 46/138 (33.3%); first imaging occurred within the Week 12 window in 34/138 (24.6%) and before the Week 12 window in 58/138 (42.0%). Accordingly, Week 12 findings were interpreted primarily as longitudinal response-associated trajectories rather than uniformly as pre-radiographic predictive signals. Episodes with PD at first imaging had worse OS compared with non-PD episodes by log-rank testing (Supplementary Figure S2).

**Figure 2:**
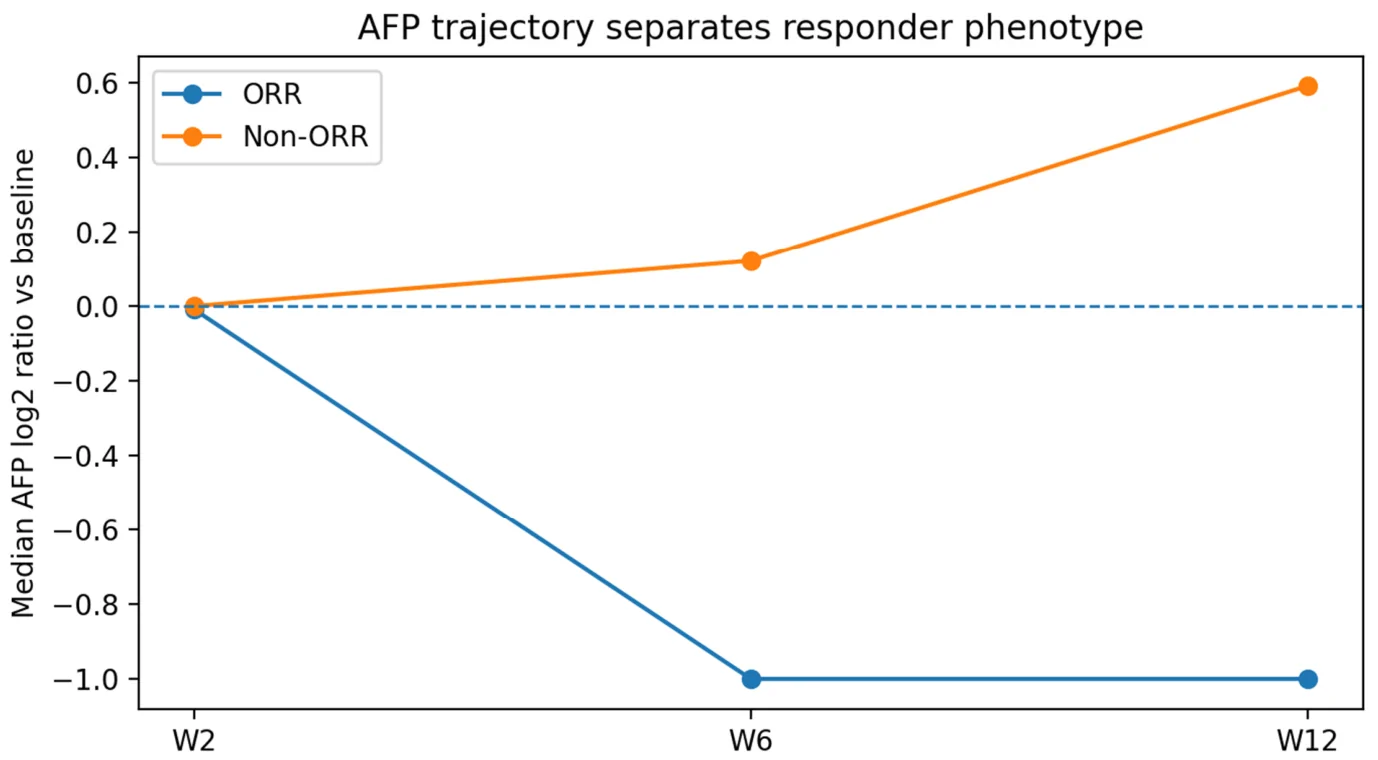
AFP trajectory by ORR status; log2 ratio > 0 indicates increase from baseline and <0 indicates decline. The blue dashed horizontal line at 0 indicates no change from baseline.

AFP trajectory provided a clinical proof-of-concept that early on-treatment change can distinguish response phenotypes. Although AFP is not an immune marker, responders demonstrated a declining AFP trajectory, while non-responders showed a rising pattern over early follow-up. This observation supports the broader study premise that dynamic biomarker behavior may provide clinically relevant response information (Figure 2).

### 3.3. Immunologic Markers Associated with Treatment Response and Overall Survival

**Figure 3:**
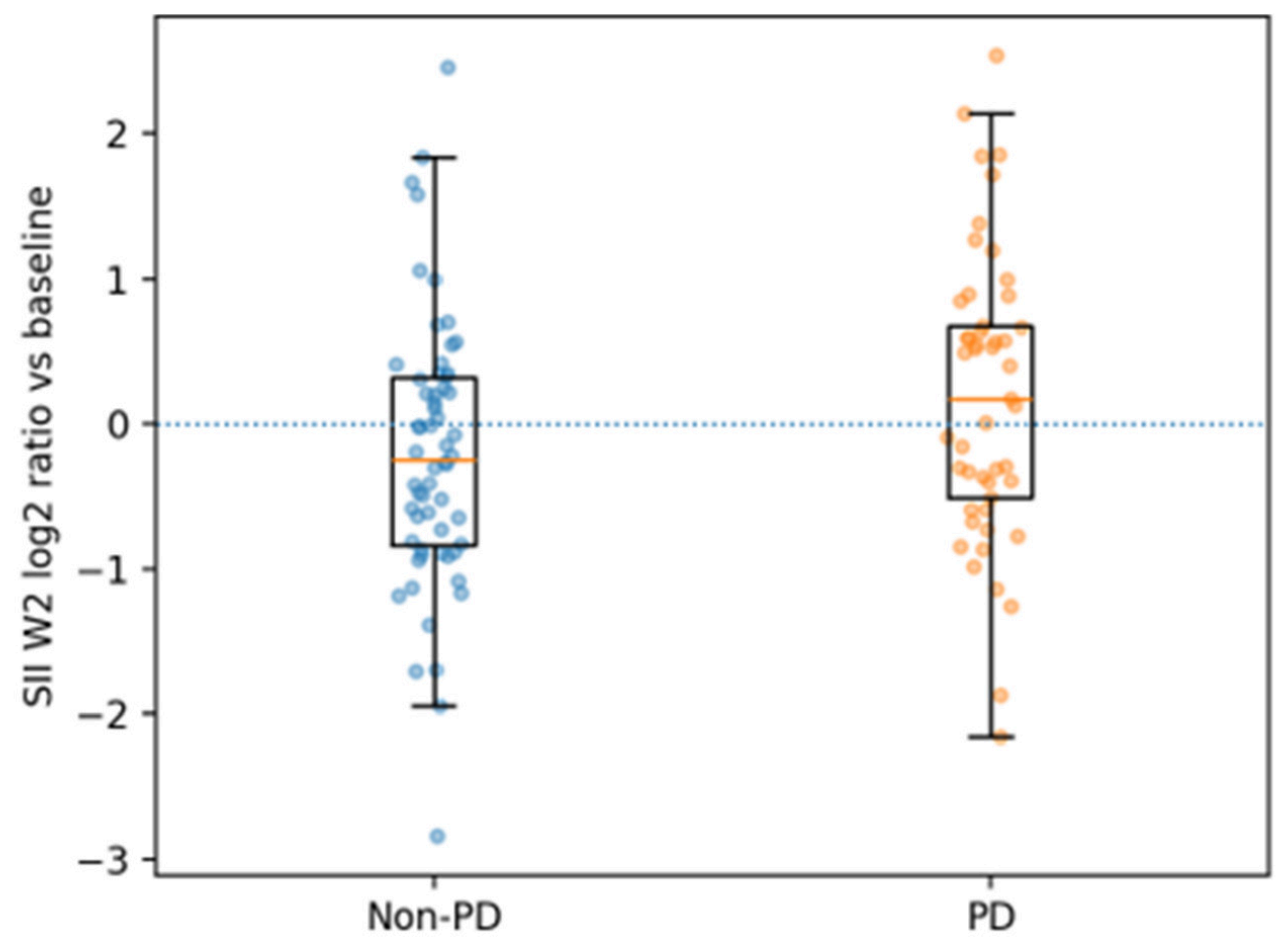
Early SII change under immunotherapy by progression status at first on-treatment imaging status. Week 2 SII log2 ratio from baseline was higher among immunotherapy-treated patients with PD at first on-treatment imaging compared with non-PD patients (median 0.17 vs. −0.24; Mann–Whitney *p* = 0.026). Values > 0 indicate increase from baseline and values < 0 indicate decline. The blue dotted horizontal line at 0 indicates no change from baseline.

**Figure 4:**
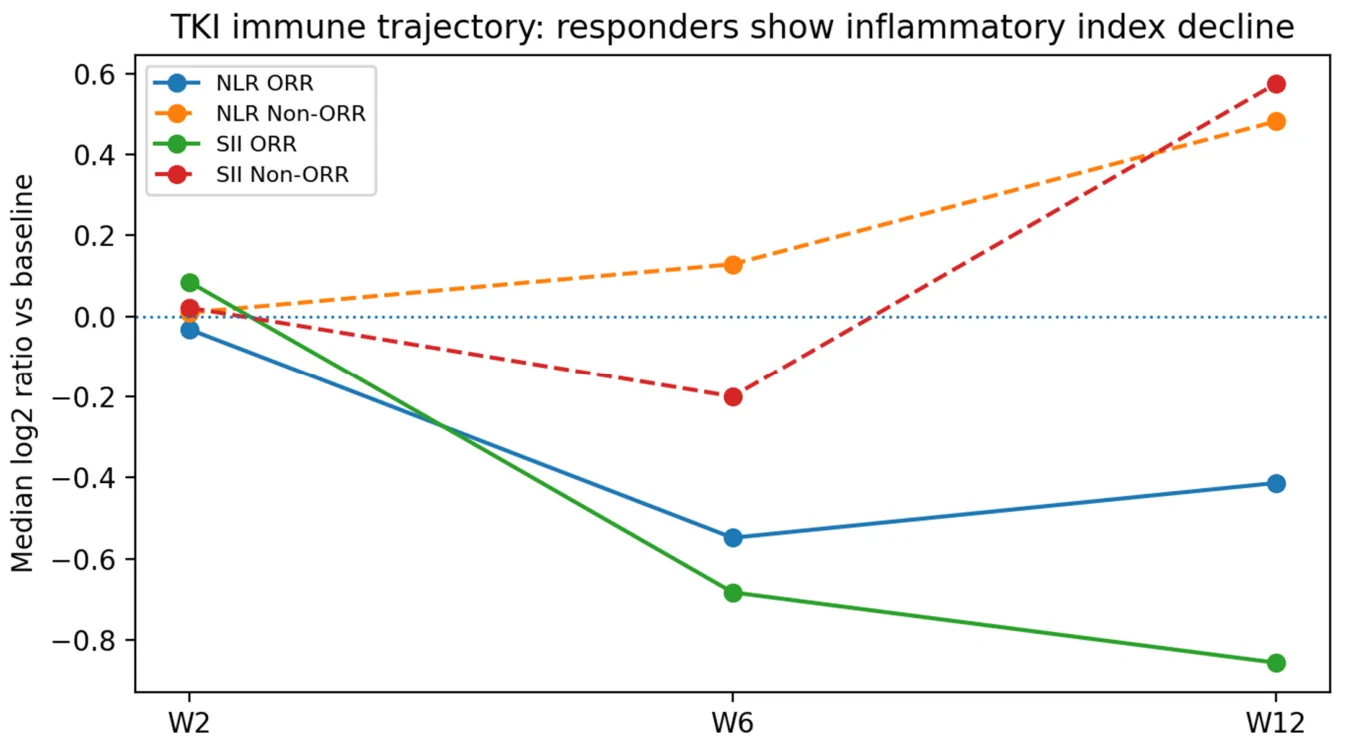
TKI immune trajectory: responders show decline in inflammatory indices over time. Values represent median log2 ratios relative to baseline; values > 0 indicate increase and values < 0 indicate decline. The blue dotted horizontal line at 0 indicates no change from baseline.

Outcome-stratified trajectories suggested that immune-cell dynamics may carry treatment-specific implication. Availability of paired longitudinal measurements varied by biomarker and timepoint. Paired SII/NLR trajectories were evaluable in 134 patients at Week 2 (109 immunotherapy, 25 TKI), 133 at Week 6 (109 immunotherapy, 24 TKI), and 130 at Week 12 (106 immunotherapy, 24 TKI). Paired AFP trajectories were available in 80, 97, and 94 patients at Weeks 2, 6, and 12, respectively. Evaluable sample sizes for the principal trajectory and sensitivity analyses are summarized in Supplementary Table S1. Among immunotherapy-treated episodes, early SII increase was associated with progression at first on-treatment imaging, with higher Week 2 SII log2 ratio in patients with PD compared with non-PD patients (median 0.17 vs. −0.24; Mann–Whitney *p* = 0.026), suggesting that early neutrophil/platelet-dominant inflammatory escalation may identify an unfavorable immunotherapy trajectory. Importantly, the Week 2 assessment window preceded first on-treatment imaging in 108/109 (99.1%) immunotherapy-treated patients contributing to this analysis. The association also remained present in a sensitivity analysis restricted to patients undergoing first imaging within 6–14 weeks (*n* = 70; median Week 2 SII log2 ratio, 0.50 for PD vs. −0.30 for non-PD; *p* = 0.016). In contrast, among TKI-treated episodes, response was associated with lymphocyte expansion and subsequent decline in NLR, SII, and neutrophil-related inflammatory burden. Progression in TKI-treated episodes showed the opposite pattern, with rising inflammatory indices over time. Together, these findings suggest that routine immune-cell indices are not sufficient for treatment selection by themselves, but they may serve as practical SOC-derived anchors for prospective immune/pharmacodynamic biomarker development (Table 3, Supplementary Table S2, Figure 3 and Figure 4).

### 3.4. Exploratory Analysis with Individual Treatment Regimen

Regimen-level analyses identified additional signals that may help prioritize prospective testing, although small subgroup sizes limit interpretation. The Week 2 SII association observed in the pooled immunotherapy cohort was not uniform across individual immunotherapy regimens. Among atezolizumab/bevacizumab-treated patients, Week 2 SII change was directionally higher in patients with PD than in those without PD (median log2 ratio, −0.22 vs. −0.38; *p* = 0.209). A similar and more pronounced pattern was observed among patients receiving single-agent PD-1/PD-L1 therapy (0.59 vs. −0.01; *p* = 0.020). In contrast, this pattern was not observed with durvalumab/tremelimumab (−0.51 vs. −0.21; *p* = 0.951). However, the overall regimen × Week 2 SII interaction was not statistically significant (*p* = 0.239), and these regimen-level analyses were limited by modest subgroup sizes. Thus, the pooled immunotherapy finding should be interpreted as an exploratory class-level association rather than evidence of a uniform regimen-specific effect. In the atezolizumab/bevacizumab group, ORR was associated with more favorable early albumin trajectories and a trend toward lymphocyte increase at Week 6. In the durvalumab/tremelimumab group, response was associated with marked AFP decline, while progression was associated with higher baseline neutrophil and NLR values. In the lenvatinib group, ORR was associated with lymphocyte expansion at Week 6 and neutrophil decline by Week 12. Representative regimen-level signals are summarized in Table 3, with the full exploratory signal list provided in Supplementary Table S3. Together, these findings suggest that the relationship between immune–inflammatory trajectories and response may differ by systemic therapy mechanism and should be prospectively tested using standardized immune, cytokine, angiogenic, and proteomic measurements.

### 3.5. Exploratory Analysis of Association Between ANA, HIV, and Outcomes

ANA status was available in 46/153 episodes; 27 known ANA episodes were ANA-positive and 19 were ANA-negative. The high proportion of unknown or untested ANA status reflects a key limitation of retrospective SOC data. Among known ANA episodes, ANA-positive immunotherapy-treated episodes had a numerically higher ORR than ANA-negative immunotherapy-treated episodes, but subgroup sizes were small and survival patterns were not clearly favorable. ANA titer analyses also remained exploratory, with low-positive and moderate/high-positive categories showing variable response patterns. These findings should be interpreted as feasibility and signal-discovery observations rather than evidence that ANA positivity selects treatment. The main implication is that ANA and SMA testing should be standardized prospectively and analyzed by treatment class as immune-context variables, not only as toxicity-risk markers. HIV infection was present in 8/153 episodes. HIV-positive episodes had numerically lower ORR and higher PD proportions than HIV-negative episodes overall, but the sample size was too small to support definitive inference. HIV status should therefore remain a prospective immune-status variable, with CD4 count and viral load captured when applicable (Supplementary Table S4).

## 4. Discussion

In this retrospective cohort of patients receiving systemic therapy for HCC, the central observation was that routine immune–inflammatory markers showed treatment-stratified trajectory patterns associated with early response phenotypes. While AFP kinetics and radiographic response provided clinical context, SII and related immune-cell indices demonstrated differing longitudinal patterns across treatment mechanisms. Formal interaction analyses supported treatment class differences for selected later neutrophil-dominant trajectories, although the interaction for the principal Week 2 SII finding was not significant. These results therefore support a treatment-context framework rather than establishing that all observed biomarker associations are intrinsically treatment-specific. Prior studies have shown that baseline SII is associated with outcomes in HCC, including in immune-based therapy settings, and recent meta-analytic evidence further supports the prognostic relevance of elevated SII across HCC cohorts [13,14,21]. Our study does not position SII as a new biomarker formula; rather, it extends the question toward whether early SII dynamics carry treatment-specific meaning. In immunotherapy-treated episodes, early SII rise was associated with progression at first on-treatment imaging, with higher Week 2 SII log2 ratio in patients with PD compared with non-PD patients. This pattern suggests that early neutrophil/platelet-dominant inflammatory escalation may reflect an unfavorable immune-response state. In contrast, TKI response was associated with lymphocyte expansion and decline in NLR, SII, and neutrophil-related inflammatory burden, suggesting a possible immune–inflammatory reversal pattern among responders. These findings remain exploratory, but they suggest that the same immune index may have different implications depending on treatment mechanism and timing.

AFP may also have relevance beyond its established role as a tumor marker. Experimental and translational studies suggest that AFP can modulate antitumor immunity through effects on dendritic cells, natural killer cells, and T-cell responses, while AFP-derived epitopes can themselves serve as targets for tumor-specific immune recognition [22]. Accordingly, the AFP trajectories observed in our cohort may reflect not only changes in tumor burden but also an evolving tumor–immune context; however, the present retrospective data do not establish a mechanistic relationship between AFP kinetics and immune response.

The regimen-level findings further support this treatment-context interpretation while underscoring the need for caution. In the atezolizumab/bevacizumab group, response was associated with more favorable early albumin trajectories and a trend toward lymphocyte expansion. In the durvalumab/tremelimumab group, response was associated with marked AFP decline, while progression was associated with higher baseline neutrophil and NLR values. In the lenvatinib group, response was associated with lymphocyte expansion by Week 6 and neutrophil decline by Week 12. These patterns are biologically plausible in the context of immune, angiogenic, and kinase-directed mechanisms, and they align with emerging molecular and single-cell data showing that systemic therapies can produce distinct immune-response programs in HCC [20]. However, the current findings should not be interpreted as treatment-selection rules. Instead, they indicate that early response biology may be captured through treatment-stratified immune–inflammatory trajectories that warrant structured prospective validation. The exploratory autoimmunity and immune-status analyses add another layer to this treatment-context framework. ANA positivity was available only in a minority of episodes, limiting inference, but ANA-positive immunotherapy-treated episodes showed a numerically higher ORR than ANA-negative episodes. This observation should not be interpreted as evidence that ANA positivity predicts benefit, especially because ANA testing was incomplete and subgroup sizes were small. Rather, it supports the concept that autoimmunity markers may reflect baseline immune tone or immune surveillance phenotypes that deserve structured evaluation beyond their usual role as toxicity-risk markers. Similarly, HIV-positive episodes were too few to support statistical inference, but the descriptive differences observed reinforce that immune competence should be considered when interpreting immune-modulating therapies. Together, these findings support a broader view in which immune-cell trajectories, tumor-marker kinetics, autoimmunity markers, and immune-status variables are complementary signals within a treatment-context response framework. The potential clinical role of an early SII trajectory should be interpreted as a monitoring signal rather than a stand-alone treatment decision tool. The present data do not support treatment discontinuation or regimen modification based on SII alone, and no clinically validated SII-change threshold was derived. If prospectively validated, however, an unfavorable early SII trajectory could serve as a low-cost flag for closer clinical and laboratory monitoring or consideration of earlier radiographic assessment, particularly when accompanied by other concerning clinical or tumor-marker changes. Treatment decisions should continue to integrate radiographic response, liver function, treatment tolerance, and overall clinical status.

From a biomarker-discovery perspective, these routine trajectories should be viewed as an accessible immunophenotypic layer rather than a substitute for molecular diagnostics. Their potential value lies in their longitudinal availability and their ability to capture evolving host–tumor inflammatory states at clinically relevant timepoints. Future prospective studies should integrate SII and related immune-cell trajectories with molecular platforms such as circulating tumor DNA kinetics, tumor or circulating-cell transcriptomic signatures, cytokine/chemokine profiling, and proteomic or immune-cell signaling assays. Such multimodal approaches may determine whether combining readily available phenotypic trajectories with molecular response markers improves early identification of treatment response or resistance and helps distinguish tumor-related immune changes from cirrhosis-associated inflammation.

Taken together, the findings nominate candidate treatment-stratified immunophenotypic trajectory signatures—including the early Week 2 SII signal and later neutrophil-dominant response patterns—for prospective molecular and clinical validation rather than establishing validated treatment-selection biomarkers.

This study has several limitations. It was retrospective and single-center, and treatment assignment was not randomized; therefore, regimen-specific differences may reflect clinical selection, liver reserve, bleeding risk, disease burden, treatment era, or eligibility for specific therapies rather than treatment mechanism alone. The immunotherapy group was also mechanistically heterogeneous, encompassing anti-PD-1/PD-L1 monotherapy, PD-L1/VEGF combination therapy, and PD-L1/CTLA-4 combination therapy. The Week 2 SII association was not directionally uniform across these regimen categories, although the formal regimen-by-SII interaction was not significant; therefore, the pooled immunotherapy signal should not be assumed to apply equally to individual regimens. Interpretation of SII in HCC also requires caution because platelet count, a core component of SII, may be influenced by cirrhosis severity, portal hypertension, hypersplenism, and hepatic reserve independently of tumor-associated immune–inflammatory activity. In the present cohort, however, the Week 2 SII association remained consistent after adjustment for Child–Pugh score and baseline platelet count and when the analysis was restricted to patients with cirrhosis. Nevertheless, direct measures of portal hypertension and hypersplenism were not systematically available in this retrospective dataset, and residual confounding by liver disease physiology cannot be excluded. Several regimen-level and ANA-defined subgroups were small, and ANA testing was missing or not performed in most episodes, so unknown ANA status should not be interpreted as true negativity. The HIV-positive subgroup was also too small for definitive conclusions. The retrospective dataset did not include matched molecular profiling, ctDNA, or standardized cytokine, angiogenic, immune-cell signaling, transcriptomic, or proteomic measurements; therefore, the present study cannot determine whether the observed peripheral immunophenotypic trajectories directly correspond to specific molecular response or resistance programs. Imaging response was categorical rather than continuous percent mRECIST viable tumor-burden change, limiting quantitative modeling of biomarker slopes against tumor-burden dynamics. In addition, response assessment relied on routine clinical imaging rather than a protocol-mandated uniform imaging modality and acquisition schedule; therefore, variation in imaging modality and timing may have introduced additional variability in radiographic response classification. Because Week 12 measurements frequently coincided with or followed first imaging, Week 12 findings should be interpreted primarily as response-associated longitudinal trajectories rather than uniformly as early predictive biomarkers. Imaging-only tumor-burden increase ≥25% was evaluated only as a descriptive subset of PD and could not be interpreted as inflammatory flare because serial IL-6 and other mechanistic inflammatory markers were unavailable. Finally, multiple biomarkers, longitudinal timepoints, outcome definitions, and regimen-level subgroup comparisons were examined without adjustment for multiplicity. Accordingly, the reported nominal *p*-values should be interpreted as hypothesis-generating rather than confirmatory, and statistically significant exploratory signals require prospective validation before clinical application. Despite these limitations, the study provides a practical real-world foundation showing that treatment-specific immune–inflammatory trajectories can be detected using available clinical data and may inform future biomarker-focused work in HCC systemic therapy.

## 5. Conclusions

This study suggests that early treatment response in HCC systemic therapy may be better understood through dynamic, treatment-specific trajectories. First imaging response and AFP kinetics provided clinically meaningful anchors, while SII and related immune-cell indices revealed distinct immune–inflammatory patterns across treatment contexts. These findings highlight the potential value of interpreting routine SOC markers not as isolated prognostic scores, but as evolving biologic signals that may reflect response, resistance, or unfavorable inflammatory trajectory. The unique contribution of this approach is its integration of routine immune-cell dynamics, tumor-marker kinetics, imaging response, and immune-context variables such as ANA and HIV status within one treatment-specific framework. Although these retrospective findings are exploratory and not sufficient to guide therapy selection on their own, they provide a practical bridge between current clinical practice and future biomarker-informed treatment strategies. By identifying which signals are detectable using available SOC data and where key biologic gaps remain, this work supports future prospective studies incorporating standardized immune, angiogenic, cytokine, autoimmunity, and proteomic measurements to refine early response prediction and improve individualized systemic therapy selection in HCC.

## Figures and Tables

**Table 1:** Baseline patient and tumor characteristics.

Characteristic	Overall (*n* = 153)	Immunotherapy (*n* = 124)	TKI (*n* = 29)
Age, years, median (IQR)	68 (63–76)	69 (64–76)	67 (61–72)
Male sex, *n* (%)	117 (76.5)	93 (75.0)	24 (82.8)
Non-Hispanic White, *n* (%)	69 (45.1)	55 (44.4)	14 (48.3)
Hispanic, *n* (%)	39 (25.5)	28 (22.6)	11 (37.9)
Non-Hispanic Asian, *n* (%)	35 (22.9)	33 (26.6)	2 (6.9)
Non-Hispanic Black, *n* (%)	7 (4.6)	5 (4.0)	2 (6.9)
Other/unknown race–ethnicity, *n* (%)	3 (2.0)	3 (2.4)	0 (0.0)
Cirrhosis, *n* (%)	130 (85.0)	103 (83.1)	27 (93.1)
HCV-SVR, *n* (%)	38 (24.8)	31 (25.0)	7 (24.1)
MASLD, *n* (%)	28 (18.3)	23 (18.5)	5 (17.2)
Alcohol-associated liver disease, *n* (%)	29 (19.0)	22 (17.7)	7 (24.1)
Active HBV, *n* (%)	22 (14.4)	19 (15.3)	3 (10.3)
Active HCV infection, *n* (%)	15 (9.8)	10 (8.1)	5 (17.2)
Other/cryptogenic/unknown risk factor, *n* (%)	33 (21.6)	29 (23.4)	4 (13.8)
BCLC A, *n* (%)	23 (15.0)	18 (14.5)	5 (17.2)
BCLC B, *n* (%)	41 (26.8)	33 (26.6)	8 (27.6)
BCLC C/D, *n* (%)	89 (58.2)	73 (58.9)	16 (55.2)
Macrovascular invasion, *n* (%)	54 (35.3)	45 (36.3)	9 (31.0)
Extrahepatic metastasis, *n* (%)	48 (31.4)	40 (32.3)	8 (27.6)
Infiltrative HCC, *n* (%)	50 (32.7)	43 (34.7)	7 (24.1)
First-line systemic therapy, *n* (%)	153 (100)	124 (100)	29 (100)
Prior LRT within 90 days, *n* (%)	56 (36.6)	43 (34.7)	13 (44.8)
Largest lesion size, cm, median (IQR)	3.8 (2.4–6.5)	4.0 (2.5–7.0)	3.5 (2.1–5.2)
CTP score, median (IQR)	6 (5–7)	6 (5–7)	7 (5–8)
MELD-Na, median (IQR)	8 (7–12)	8 (7–12)	9 (7–11)
Albumin, g/dL, median (IQR)	3.6 (3.1–4.0)	3.6 (3.1–4.0)	3.7 (3.1–4.0)
AFP, ng/mL, median (IQR)	130 (8–1259)	280 (11–2259)	26 (4–275)
Baseline NLR, median (IQR)	3.4 (2.3–5.3)	3.3 (2.2–5.3)	3.6 (2.4–4.1)
Baseline SII, median (IQR)	510.2 (289.0–988.0)	517.1 (286.0–987.6)	438.2 (299.6–980.6)
ANA known/tested, *n* (%)	46 (30.1)	36 (29.0)	10 (34.5)
ANA-positive among known, *n*/*N* (%)	27/46 (58.7)	21/36 (58.3)	6/10 (60.0)
HIV infection, *n* (%)	8 (5.2)	7 (5.6)	1 (3.4)

Values are *n* (%) unless otherwise indicated. Liver disease risk factors are not mutually exclusive. ANA positivity percentage is calculated among episodes with known/tested ANA status.

**Table 2:** First imaging response by therapy class and regimen.

Group	*n*	CR	PR	SD	PD	ORR *n* (%)	DCR *n* (%)	PD *n* (%)	PD with ≥25% Tumor-Burden Increase *n*/*N* (%)
Atezo/Bev	46	4	9	11	22	13/46 (28.3%)	24/46 (52.2%)	22/46 (47.8%)	8/22 (36.4%)
Durva/Treme	31	0	12	8	11	12/31 (38.7%)	20/31 (64.5%)	11/31 (35.5%)	5/11 (45.5%)
Nivolumab	27	2	3	9	13	5/27 (18.5%)	14/27 (51.9%)	13/27 (48.1%)	5/13 (38.5%)
Lenvatinib	15	1	3	4	7	4/15 (26.7%)	8/15 (53.3%)	7/15 (46.7%)	4/7 (57.1%)
Sorafenib	13	1	0	9	3	1/13 (7.7%)	10/13 (76.9%)	3/13 (23.1%)	0/3 (0.0%)
Pembrolizumab	9	0	3	1	5	3/9 (33.3%)	4/9 (44.4%)	5/9 (55.6%)	0/5 (0.0%)
Atezolizumab alone	7	1	1	1	4	2/7 (28.6%)	3/7 (42.9%)	4/7 (57.1%)	1/4 (25.0%)
Durvalumab alone	4	0	1	1	2	1/4 (25.0%)	2/4 (50.0%)	2/4 (50.0%)	1/2 (50.0%)
Regorafenib	1	0	0	1	0	0/1 (0.0%)	1/1 (100.0%)	0/1 (0.0%)	N/A
Immunotherapy	124	7	29	31	57	36/124 (29.0%)	67/124 (54.0%)	57/124 (46.0%)	20/57 (35.1%)
TKI	29	2	3	14	10	5/29 (17.2%)	19/29 (65.5%)	10/29 (34.5%)	4/10 (40.0%)
Overall	153	9	32	45	67	41/153 (26.8%)	86/153 (56.2%)	67/153 (43.8%)	24/67 (35.8%)

PD with ≥25% tumor-burden increase is reported using PD cases as the denominator. This exploratory variable was not considered a separate response category and should not be interpreted as protocol-defined paradoxical flare because contemporaneous IL-6 data were unavailable.

**Table 3:** Key treatment-stratified immune/routine trajectory signals associated with early response phenotypes.

Treatment Context	Outcome Comparison	Marker or Trajectory	Group 1, Median (IQR)	Group 2, Median (IQR)	*p*-Value	Interpretation
Immunotherapy	PD vs. non-PD	Week 2 SII log2 ratio	PD: 0.17	Non-PD: −0.24	0.026	Early SII rise was associated with progression at first on-treatment imaging.
Atezolizumab/bevacizumab	ORR vs. no ORR	Albumin Week 2 log2 ratio	ORR: 0.04 (−0.02 to 0.09)	No ORR: −0.07 (−0.13 to 0.00)	0.011	Responders showed a more favorable early albumin trajectory.
Atezolizumab/bevacizumab	ORR vs. no ORR	Lymphocyte Week 6 log2 ratio	ORR: 0.24 (0.02 to 0.42)	No ORR: −0.03 (−0.44 to 0.22)	0.077	Responders showed a trend toward lymphocyte expansion.
Atezolizumab/bevacizumab	PD vs. non-PD	NLR Week 2 log2 ratio	PD: 0.39 (−0.45 to 0.60)	Non-PD: −0.31 (−0.68 to 0.25)	0.089	Progression was associated with a trend toward early inflammatory-index rise.
Durvalumab/tremelimumab	ORR vs. no ORR	AFP Week 12 log2 ratio	ORR: −4.83 (−6.66 to −1.00)	No ORR: 0.80 (−0.07 to 1.52)	0.016	Response was associated with marked AFP decline.
Durvalumab/tremelimumab	PD vs. non-PD	Baseline neutrophil count	PD: 3.86 (3.12 to 6.70)	Non-PD: 2.69 (1.96 to 4.03)	0.033	Progression was associated with higher baseline neutrophil burden.
Lenvatinib	ORR vs. no ORR	Lymphocyte Week 6 log2 ratio	ORR: 0.74 (0.57 to 1.45)	No ORR: 0.13 (−0.17 to 0.36)	0.014	TKI response was associated with lymphocyte expansion.
Lenvatinib	ORR vs. no ORR	Neutrophil Week 12 log2 ratio	ORR: −1.15 (−1.35 to −0.78)	No ORR: 0.28 (−0.07 to 0.73)	0.020	TKI response was associated with declining neutrophil-related inflammatory burden.

Note: Representative signals were selected for main display based on relevance to the manuscript’s treatment-specific immune–inflammatory trajectory hypothesis. Full exploratory signal lists are provided in Supplementary Tables S2 and S3. ORR indicates complete or partial response; PD indicates progressive disease at first on-treatment imaging. Trajectory variables are shown as log2 ratio relative to baseline, where values > 0 indicate increase from baseline and values < 0 indicate decline. *p*-values were calculated using Mann–Whitney rank-based testing and are interpreted descriptively.

## Data Availability

The data generated and analyzed in this study are not publicly available due to patient privacy and institutional restrictions. De-identified data supporting the findings may be available from the corresponding author upon reasonable request and with required institutional approvals.

## Supplementary Materials

The following supporting information can be downloaded at https://www.mdpi.com/article/10.3390/diagnostics16203329/s1, Table S1: Evaluable sample sizes and robustness analyses for key immune-inflammatory trajectory findings; Table S2: Selected immune/routine trajectory signals by treatment class and outcome (*p* < 0.10); Table S3: Regimen-specific immune/routine marker signals (*p* < 0.10); Table S4: Response and survival by ANA positivity among known ANA episodes; Figure S1: First on-treatment imaging response distribution by therapy class; Figure S2: Survival probability by progression status at first on-treatment imaging status.
