# Supplementary material for: Treatment-Stratified Immune–Inflammatory Trajectories and Early Response in Patients with Hepatocellular Carcinoma Receiving Systemic Therapy
Source: Diagnostics (Basel). 2026 Oct 13;16(20):3329. doi: 10.3390/diagnostics16203329 (PMC13703371; doi:10.3390/diagnostics16203329)
Supplement: Supplementary file 1 [file diagnostics-16-03329-s001.zip › Supplementary Table S2.pdf]

Supplementary Table S2. Selected immune/routine trajectory signals by treatment class and outcome (p<0.10)

Analysis group	Feature	Deceased, n	Deceased, median (IQR)	Alive, n	Alive, median (IQR)	p value
All episodes	SII W12 log2 ratio	87	0.31 (-0.46-0.82)	31	-0.36 (-0.88-0.19)	0.004
All episodes	Albumin W12 log2 ratio	90	-0.08 (-0.24-0.00)	33	-0.03 (-0.07-0.10)	0.005
All episodes	Neutrophil W12 log2 ratio	87	0.22 (-0.15-0.71)	31	-0.13 (-0.32-0.18)	0.008
All episodes	AFP W2 log2 ratio	52	0.06 (-0.11-0.81)	21	-0.35 (-1.00-0.09)	0.016
All episodes	NLR W12 log2 ratio	87	0.23 (-0.23-0.76)	31	-0.21 (-0.57-0.18)	0.019
All episodes	Baseline hsCRP	21	11.10 (7.30-19.00)	9	1.90 (0.50-4.80)	0.019
All episodes	Bilirubin W12 log2 ratio	90	0.00 (0.00-0.60)	32	0.00 (0.00-0.00)	0.020
All episodes	AFP W6 log2 ratio	63	0.09 (-1.00-0.73)	27	-0.43 (-2.44-0.05)	0.029
All episodes	SII W6 log2 ratio	89	0.06 (-0.57-0.80)	31	-0.28 (-0.75-0.05)	0.031
All episodes	SII W2 log2 ratio	89	-0.01 (-0.61-0.57)	33	-0.42 (-1.09-0.20)	0.043
All episodes	NLR W6 log2 ratio	89	0.17 (-0.50-0.65)	31	-0.14 (-0.70-0.25)	0.046
All episodes	AFP W12 log2 ratio	60	0.42 (-0.62-1.51)	27	-0.42 (-5.30-1.03)	0.047
All episodes	Monocyte W12 log2 ratio	87	0.17 (-0.17-0.50)	31	-0.11 (-0.33-0.27)	0.051
All episodes	Bilirubin W6 log2 ratio	94	0.00 (0.00-0.55)	34	0.00 (0.00-0.10)	0.066
All episodes	MLR W12 log2 ratio	87	0.13 (-0.36-0.46)	31	-0.20 (-0.51-0.27)	0.073
All episodes	LMR W12 log2 ratio	87	-0.13 (-0.46-0.36)	31	0.20 (-0.27-0.51)	0.073
All episodes	NLR W2 log2 ratio	89	0.06 (-0.52-0.64)	33	-0.20 (-0.68-0.30)	0.097
Immunotherapy	AFP W2 log2 ratio	43	0.10 (-0.01-0.85)	20	-0.40 (-1.10-0.02)	0.003
Immunotherapy	AFP W6 log2 ratio	51	0.19 (-0.74-0.98)	23	-0.46 (-4.35--0.12)	0.004
Immunotherapy	Baseline hsCRP	18	10.65 (6.67-18.88)	7	1.90 (0.45-4.45)	0.012
Immunotherapy	SII W12 log2 ratio	69	0.29 (-0.47-0.82)	28	-0.37 (-0.85-0.29)	0.014
Immunotherapy	Neutrophil W12 log2 ratio	69	0.22 (-0.17-0.63)	28	-0.14 (-0.35-0.20)	0.019
Immunotherapy	Albumin W12 log2 ratio	71	-0.07 (-0.25-0.00)	29	-0.03 (-0.07-0.10)	0.021
Immunotherapy	AFP W12 log2 ratio	50	0.37 (-0.65-1.09)	24	-0.79 (-5.84-0.70)	0.034
Immunotherapy	Monocyte W12 log2 ratio	69	0.21 (-0.18-0.51)	28	-0.12 (-0.33-0.27)	0.038

Values compare patients who were deceased versus alive at last follow-up. Trajectory variables are shown as log2 ratio relative to baseline, where values >0 indicate increase from baseline and values <0 indicate decline. P values were calculated using Mann-Whitney rank-based testing and are interpreted descriptively.
