# Supplementary material for: Treatment-Stratified Immune–Inflammatory Trajectories and Early Response in Patients with Hepatocellular Carcinoma Receiving Systemic Therapy
Source: Diagnostics (Basel). 2026 Oct 13;16(20):3329. doi: 10.3390/diagnostics16203329 (PMC13703371; doi:10.3390/diagnostics16203329)
Supplement: Supplementary file 1 [file diagnostics-16-03329-s001.zip › Supplementary Table S3.pdf]

Supplementary Table S3. Regimen-specific immune/routine marker signals (p<0.10)

Analysis group	Outcome evaluated	Feature	Yes, n	Yes, median (IQR)	No, n	No, median (IQR)	p value
Atezo/Bev	ORR	Albumin W2 log2 ratio	11	0.04 (-0.02-0.09)	26	-0.07 (-0.13-0.00)	0.011
Atezo/Bev	ORR	Albumin W6 log2 ratio	12	0.00 (-0.07-0.12)	27	-0.10 (-0.19-0.00)	0.027
Atezo/Bev	ORR	Lymphocyte W6 log2 ratio	12	0.24 (0.02-0.42)	25	-0.03 (-0.44-0.22)	0.077
Atezo/Bev	ORR	AFP W12 log2 ratio	10	-0.75 (-3.51-0.23)	17	0.10 (-0.36-1.07)	0.079
Atezo/Bev	PD	Bilirubin W12 log2 ratio	18	0.00 (0.00-0.00)	22	0.26 (0.00-0.75)	0.036
Atezo/Bev	PD	Baseline AFP	17	695.00 (27.00-1984.00)	22	18.50 (4.00-831.75)	0.086
Atezo/Bev	PD	NLR W2 log2 ratio	16	0.39 (-0.45-0.60)	20	-0.31 (-0.68-0.25)	0.089
Durva/Treme	ORR	AFP W12 log2 ratio	10	-4.83 (-6.66--1.00)	16	0.80 (-0.07-1.52)	0.016
Durva/Treme	ORR	Bilirubin W12 log2 ratio	12	0.00 (-0.25-0.00)	18	0.00 (0.00-0.29)	0.018
Durva/Treme	ORR	AFP W6 log2 ratio	11	-3.75 (-6.11--0.39)	17	0.42 (-0.25-1.25)	0.024
Durva/Treme	ORR	Albumin W6 log2 ratio	12	-0.11 (-0.20--0.10)	19	0.00 (-0.11-0.11)	0.041
Durva/Treme	ORR	Platelet W6 log2 ratio	11	-0.16 (-0.31--0.08)	19	0.02 (-0.19-0.09)	0.048
Durva/Treme	ORR	AFP W2 log2 ratio	10	-0.92 (-1.07--0.03)	14	0.05 (-0.14-0.72)	0.057
Durva/Treme	ORR	Baseline Monocyte	12	0.37 (0.29-0.60)	19	0.54 (0.42-0.73)	0.077
Durva/Treme	ORR	Baseline Neutrophil	12	2.24 (1.92-3.58)	19	3.86 (2.83-5.03)	0.078
Durva/Treme	PD	Baseline Neutrophil	11	3.86 (3.12-6.70)	20	2.69 (1.96-4.03)	0.033
Durva/Treme	PD	Baseline NLR	11	4.77 (2.36-7.92)	20	2.92 (1.70-4.02)	0.066
Durva/Treme	PD	Platelet W6 log2 ratio	11	0.05 (-0.13-0.11)	19	-0.11 (-0.28-0.00)	0.078
Durva/Treme	PD	MLR W12 log2 ratio	11	-0.43 (-0.52-0.06)	18	0.13 (-0.21-0.35)	0.084
Durva/Treme	PD	LMR W12 log2 ratio	11	0.43 (-0.06-0.52)	18	-0.13 (-0.35-0.21)	0.084
Durva/Treme	PD	AFP W2 log2 ratio	7	0.30 (0.00-0.61)	17	-0.18 (-1.00-0.09)	0.086
Durva/Treme	PD	NLR W12 log2 ratio	11	-0.42 (-0.57-0.04)	18	0.26 (-0.37-1.02)	0.092
Durva/Treme	PD	AFP W12 log2 ratio	10	0.80 (0.16-1.21)	16	-1.00 (-5.84-1.51)	0.097
Lenvatinib	ORR	Lymphocyte W6 log2 ratio	3	0.74 (0.57-1.45)	10	0.13 (-0.17-0.36)	0.014
Lenvatinib	ORR	Neutrophil W12 log2 ratio	4	-1.15 (-1.35--0.78)	9	0.28 (-0.07-0.73)	0.020

Note: “Yes” and “No” refer to the outcome listed in the “Outcome evaluated” column. For ORR rows, “Yes” indicates patients with objective response and “No” indicates patients without objective response. For PD rows, “Yes” indicates patients with progressive disease at first on-treatment imaging and “No” indicates patients without progressive disease. Trajectory variables are shown as log2 ratio relative to baseline, where values >0 indicate increase from baseline and values <0 indicate decline. P values were calculated using Mann–Whitney rank-based testing and are interpreted descriptively.
