# Supplementary material for: Treatment-Stratified Immune–Inflammatory Trajectories and Early Response in Patients with Hepatocellular Carcinoma Receiving Systemic Therapy
Source: Diagnostics (Basel). 2026 Oct 13;16(20):3329. doi: 10.3390/diagnostics16203329 (PMC13703371; doi:10.3390/diagnostics16203329)
Supplement: Supplementary file 1 [file diagnostics-16-03329-s001.zip › Supplementary Table S4.pdf]

Supplementary Table S4. Response and survival by ANA positivity among known ANA episodes

Group	ANA status	n	ORR n (%)	PD n (%)	DCR n (%)	Death n/N (%)	Median OS days
All known ANA	ANA positive	27	8/27 (29.6%)	12/27 (44.4%)	15/27 (55.6%)	20/26 (76.9%)	174.0
All known ANA	ANA negative	19	4/19 (21.1%)	8/19 (42.1%)	11/19 (57.9%)	13/19 (68.4%)	385.0
Known ANA: Immunotherapy	ANA positive	21	8/21 (38.1%)	8/21 (38.1%)	13/21 (61.9%)	15/20 (75.0%)	168.0
Known ANA: Immunotherapy	ANA negative	15	4/15 (26.7%)	7/15 (46.7%)	8/15 (53.3%)	9/15 (60.0%)	406.0
Known ANA: TKI	ANA positive	6	0/6 (0.0%)	4/6 (66.7%)	2/6 (33.3%)	5/6 (83.3%)	243.0
Known ANA: TKI	ANA negative	4	0/4 (0.0%)	1/4 (25.0%)	3/4 (75.0%)	4/4 (100.0%)	167.5
