# Supplementary material for: Treatment-Stratified Immune–Inflammatory Trajectories and Early Response in Patients with Hepatocellular Carcinoma Receiving Systemic Therapy
Source: Diagnostics (Basel). 2026 Oct 13;16(20):3329. doi: 10.3390/diagnostics16203329 (PMC13703371; doi:10.3390/diagnostics16203329)
Supplement: Supplementary file 1 [file diagnostics-16-03329-s001.zip › Supplementary Figure S2.pdf]

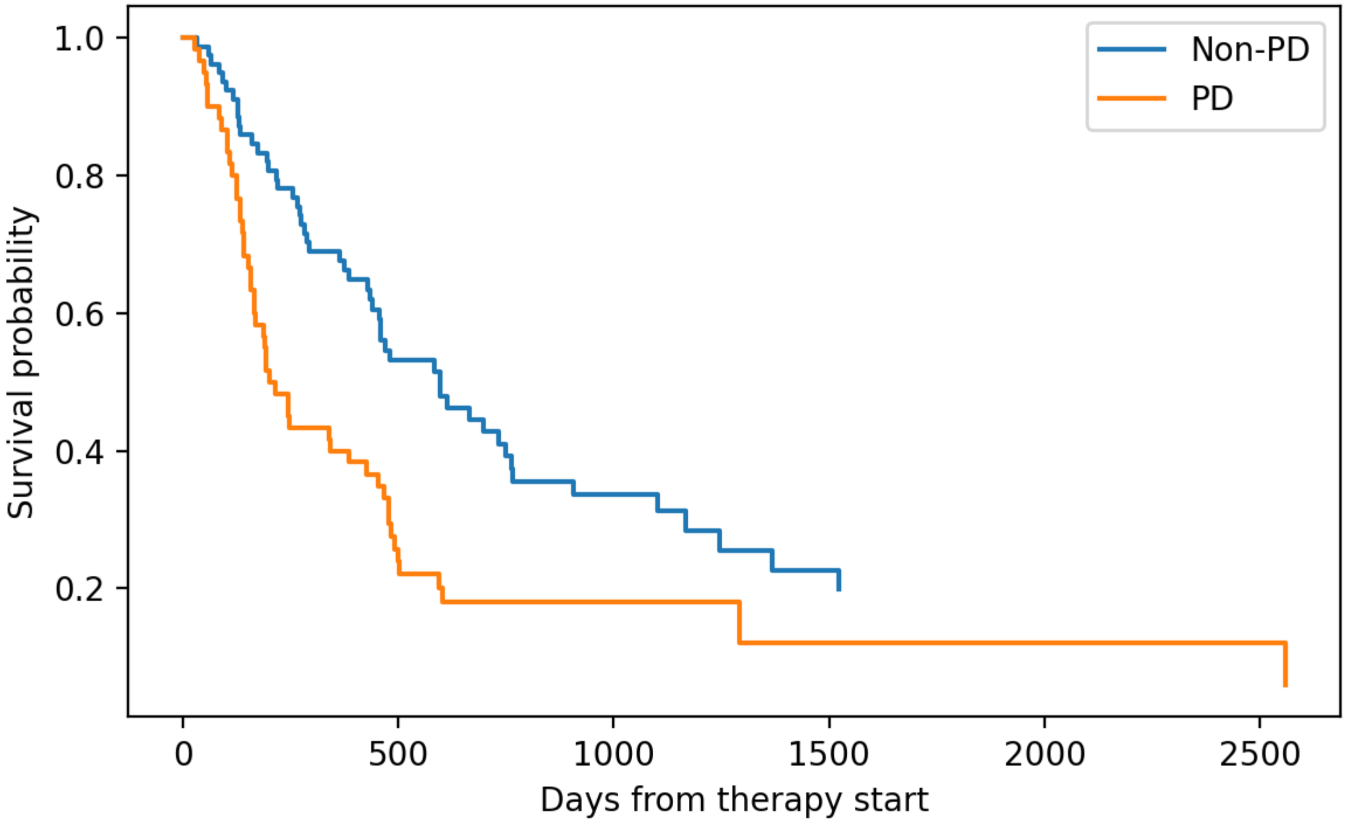

Supplementary Figure S2. Survival probability by progression status at first on-treatment imaging status.
