# Supplementary material for: Treatment-Stratified Immune–Inflammatory Trajectories and Early Response in Patients with Hepatocellular Carcinoma Receiving Systemic Therapy
Source: Diagnostics (Basel). 2026 Oct 13;16(20):3329. doi: 10.3390/diagnostics16203329 (PMC13703371; doi:10.3390/diagnostics16203329)
Supplement: Supplementary file 1 [file diagnostics-16-03329-s001.zip › Supplementary Table S1.pdf]

Supplementary Table S1. Evaluable sample sizes and robustness analyses for key immune-inflammatory trajectory findings

Analysis domain	Analysis / population	n	Descriptive result or effect estimate	p value	Interpretation
**A. Cohort composition and evaluable longitudinal measurements**					
**Treatment line**	Index systemic-therapy episodes	153	153/153 (100%) first-line; no prior systemic HCC therapy before Day 0	—	The full analytic cohort represents first-line treatment.
**Paired SII/NLR**	Week 2 trajectory	134	109 immunotherapy; 25 TKI	—	Paired baseline and Week 2 values available.
**Paired SII/NLR**	Week 6 trajectory	133	109 immunotherapy; 24 TKI	—	Paired baseline and Week 6 values available.
**Paired SII/NLR**	Week 12 trajectory	130	106 immunotherapy; 24 TKI	—	Paired baseline and Week 12 values available.
**Paired AFP**	Week 2 trajectory	80	69 immunotherapy; 11 TKI	—	Paired baseline and Week 2 AFP values available.
**Paired AFP**	Week 6 trajectory	97	80 immunotherapy; 17 TKI	—	Paired baseline and Week 6 AFP values available.
**Paired AFP**	Week 12 trajectory	94	79 immunotherapy; 15 TKI	—	Paired baseline and Week 12 AFP values available.
**B. Imaging timing and temporal sensitivity analyses**					
**First imaging timing**	Overall cohort	153	Median 79 days (IQR 60-101) from Day 0	—	Imaging followed routine clinical care, not a study-mandated schedule.
**First imaging timing**	Immunotherapy	124	Median 79 days (IQR 61-99)	—	Similar median timing to the overall cohort.
**First imaging timing**	TKI	29	Median 79 days (IQR 55-111)	—	Similar median timing, with wider dispersion.
**Window vs imaging**	Week 6 laboratory window	142	Entirely before imaging: 119/142 (83.8%); overlaps imaging: 11/142 (7.7%); imaging before window: 12/142 (8.5%)	—	Week 6 was predominantly pre-radiographic.
**Window vs imaging**	Week 12 laboratory window	138	Entirely before imaging: 46/138 (33.3%); overlaps imaging: 34/138 (24.6%); imaging before window: 58/138 (42.0%)	—	Week 12 is best interpreted as a longitudinal response-associated timepoint, not uniformly pre-radiographic.
**Week 2 timing**	Immunotherapy patients in principal Week 2 SII analysis	109	Week 2 window entirely preceded imaging in 108/109 (99.1%); 1/109 overlapped	—	Supports interpretation of the Week 2 SII signal as early and predominantly pre-radiographic.
**Imaging-window sensitivity**	Immunotherapy; first imaging restricted to 6-14 weeks	70	Week 2 SII log2 ratio: PD 0.50 vs non-PD -0.30	0.016	The principal Week 2 SII association remained present with clinically timed first imaging.
**C. Principal effect estimate and treatment-by-marker interaction analyses**					
**Principal Week 2 SII**	Immunotherapy; progression outcome	109	OR 1.55 (95% CI 1.02-2.34) per 1-unit increase in Week 2 SII log2 ratio	0.038	Higher early SII change was associated with greater odds of progression.
**Treatment-class interaction**	Week 2 SII -> PD	134	Interaction OR 1.17 (95% CI 0.44-3.09)	0.757	No evidence that the Week 2 SII association differed significantly between treatment classes.
**Treatment-class interaction**	Week 6 lymphocyte change -> ORR	133	Interaction OR 24.89 (95% CI 0.50-1239.96)	0.107	Large imprecision; interaction not statistically significant.
**Treatment-class interaction**	Week 12 neutrophil change -> ORR	130	Interaction OR 0.084 (95% CI 0.008-0.903)	0.041	Supports a treatment-class difference for this later response-associated trajectory.
**Treatment-class interaction**	Week 12 NLR change -> ORR	130	Interaction OR 0.172 (95% CI 0.031-0.962)	0.045	Supports a treatment-class difference for this later response-associated trajectory.
**Treatment-class interaction**	Week 12 SII change -> ORR	130	Interaction OR 0.298 (95% CI 0.075-1.186)	0.086	Nonsignificant trend only.
**D. Week 2 SII by major immunotherapy regimen**					
**Regimen-stratified**	Atezolizumab/bevacizumab	36	PD median -0.22 vs non-PD -0.38	0.209	Directionally consistent with the pooled immunotherapy association, but not significant.
**Regimen-stratified**	Durvalumab/tremelimumab	31	PD median -0.51 vs non-PD -0.21	0.951	Directional pattern not observed.
**Regimen-stratified**	Single-agent PD-1/PD-L1	42	PD median 0.59 vs non-PD -0.01	0.020	Higher Week 2 SII in PD; exploratory subgroup association.
**Regimen x SII interaction**	Global comparison across the three immunotherapy regimen categories	109	Likelihood-ratio interaction test	0.239	No statistically significant evidence of regimen-level heterogeneity in the SII-progression association.

Analysis domain	Analysis / population	n	Descriptive result or effect estimate	p value	Interpretation
**E. Hepatic-reserve and cirrhosis sensitivity analyses of Week 2 SII**					
**Adjusted model**	Immunotherapy; adjusted for baseline Child-Pugh score	109	Adjusted OR 1.58 (95% CI 1.04-2.41)	0.034	Week 2 SII association persisted after accounting for hepatic reserve.
**Adjusted model**	Immunotherapy; adjusted for baseline MELD-Na	109	Adjusted OR 1.56 (95% CI 1.03-2.37)	0.038	Consistent result using an alternative hepatic-reserve measure.
**Adjusted model**	Immunotherapy; adjusted for Child-Pugh score + baseline platelet count	109	Adjusted OR 1.65 (95% CI 1.07-2.55)	0.025	Association persisted after accounting for hepatic reserve and baseline platelet count.
**Cirrhosis-restricted**	Immunotherapy patients with cirrhosis	91	Week 2 SII log2 ratio: PD 0.41 vs non-PD -0.24	0.024	Association remained present when restricted to cirrhosis.
**Cirrhosis-restricted adjusted**	Cirrhosis only; adjusted for Child-Pugh score	91	Adjusted OR 1.63 (95% CI 1.03-2.57)	0.036	Association persisted after hepatic-reserve adjustment within cirrhosis.
**Cirrhosis-restricted adjusted**	Cirrhosis only; adjusted for Child-Pugh score + baseline platelet count	91	Adjusted OR 1.74 (95% CI 1.08-2.80)	0.023	Association persisted after additional platelet adjustment within cirrhosis.

Notes: SII, systemic immune-inflammation index; NLR, neutrophil-to-lymphocyte ratio; AFP, alpha-fetoprotein; TKI, tyrosine kinase inhibitor; PD, progressive disease at first on-treatment imaging; ORR, objective response rate; IQR, interquartile range; OR, odds ratio; CI, confidence interval. Trajectory measures are log2 ratios relative to baseline. For imaging-timing rows, an assessment window was classified as entirely before imaging when the complete prespecified laboratory window preceded the imaging date; overlap indicates that imaging occurred within the laboratory window. Interaction ORs are from logistic regression models containing treatment class, continuous biomarker log2 ratio, and their interaction term. Regimen-level and sensitivity analyses are exploratory; nominal p values were not adjusted for multiplicity and should be interpreted as hypothesis-generating.
